# Body Image as a Mediating Mechanism in the Association Between Media Literacy and Acceptance of Cosmetic Surgery Among Young Adults

**DOI:** 10.1007/s00266-026-06069-7

**Published:** 2026-07-07

**Authors:** Güleser Ada, Ebru Cirban Ekrem

**Affiliations:** 1 Department of Midwifery, Faculty of Health Sciences, Bartin University, Bartin, Turkey; 2 Department of Gynecology and Obstetrics Nursing, Faculty of Health Sciences, Bartin University, Bartin, Turkey

**Keywords:** Media literacy, Body image, Cosmetic surgery, Mediation

## Abstract

**Background:**

The increasing use of social media has significantly influenced how young people perceive their appearance and attitudes toward aesthetic interventions.

**Objective:**

This study aimed to examine the relationship between media literacy and acceptance of cosmetic surgery among young adults and to test the mediating role of body image.

**Methods:**

This descriptive cross sectional study was conducted in Türkiye between December 2025 and February 2026 with 394 young adults aged 18–24 years. Data were collected online using the Introductory Information Form, Media Literacy Scale, Body Image Scale, and Acceptance of Cosmetic Surgery Scale. Pearson’s correlation analysis was used to examine relationships between variables. Mediation analysis was performed using PROCESS Macro (Model 4) with 5000 bootstrap samples.

**Results:**

The mean age of participants was 22.32±4.47 years; 76.6% were female and 91.4% had a university education. Media literacy was significantly and negatively associated with body image (*β* = − 0.300, *p* < .001), indicating that higher media literacy was associated with lower levels of negative body image. Body image was significantly and positively associated with acceptance of cosmetic surgery (*β* = 0.280, *p* < .001). Mediation analysis revealed that body image showed a partial mediating role in the association between media literacy and acceptance of cosmetic surgery.

**Conclusion:**

Higher media literacy was associated with lower levels of negative body image and indirectly influenced attitudes toward cosmetic surgery. Enhancing media literacy may support healthier perceptions of body image among young adults.

**Level of Evidence V:**

This journal requires that authors assign a level of evidence to each article. For a full description of these Evidence-Based Medicine ratings, please refer to the Table of Contents or the online Instructions to Authors www.springer.com/00266.

## Introduction

Today, an array of social media platforms serves as one of the most powerful sociocultural arenas that produce, circulate, and normalize ideals of appearance. Social media websites, particularly Instagram and Snapchat, emphasize visual self-presentation and provide instances for ongoing engagement with beauty content. In doing so, they shape how people present themselves to others, how they assess their own bodies, and how they think about aesthetic intervention. According to studies by AlBahlal et al. [1], Arab et al. [2] and Eldaly and Mashaly [3], continued exposure to idealized images may amplify dissatisfaction with appearance and increase the likelihood of using cosmetic procedures.

Social media functions through highly interactive processes, unlike traditional media. Users do not just consume content. They are active participants in constructing and circulating digital content through comparing, responding, and editing, as well as through more theatrical displays or self-monitoring. They engage with content involving both themselves and others. As a result, peer images, celebrity aesthetics, filters, and appearance-based feedback become ingrained in everyday self-evaluation. Filtered, curated, or cosmetically enhanced images appear to increase interest in aesthetic procedures and alter perceptions of what is normal or desirable [4, 5].

At the psychological level, body dissatisfaction and social appearance anxiety are among the many factors shaping attitudes toward physical alteration. Social appearance anxiety refers to concern about how one’s appearance may be judged by others in social settings. Such concerns are especially salient in young adulthood, when identity formation, social visibility, and bodily self-awareness remain highly active. Previous studies indicate that dissatisfaction with specific body areas is increasing in both women and men and is associated with more favorable views of cosmetic surgery [1, 6–8].

Body image itself is a multidimensional construct encompassing perceptions, feelings, evaluations, and beliefs about the body [9]. It includes perceptions of weight, size, shape, appearance, and bodily functioning. A positive body image has been associated with stronger self-esteem, lower depressive symptomatology, greater social engagement, and healthier behavioral outcomes [10]. By contrast, negative body image has been linked to a wide range of adverse outcomes, including disordered eating, anxiety, depressive symptoms, and self-harm [11–14]. Within social media environments, appearance-based comparison with peers, influencers, and celebrities may facilitate the internalization of unrealistic beauty norms, thereby intensifying dissatisfaction with one’s own body [15, 16]. Such processes have also been associated with greater acceptance of cosmetic procedures [17–19].

One key theoretical framework for understanding these processes is the Tripartite Influence Model [20]. The model states that body image is influenced by three main sociocultural agents: family, peers, and media. Body image is not influenced by these sources only directly. However, they do promote social comparison and the internalization of appearance ideals. In today’s digital culture, social media may amplify both processes by triggering constant visual access and automatic evaluative feedback [21].

The young adult age period (18–24 years) is of particular importance in this respect. It is these years during which social identity, bodily self-perception, and interpersonal positioning are often negotiated with particular intensity [22, 23]. Young adults are frequently exposed to appearance-based comparison and beauty-related pressure as they actively utilize social media [9, 24]. This age group is more likely to use social media. While the literature has focused largely on women, the evidence suggests that body dissatisfaction and desire for aesthetic intervention are also increasing in men [24]. It is pertinent to all genders equally.

In this greater context, media literacy becomes particularly important. The ability to critically assess, analyze, interpret, evaluate, and respond to media messages is what we call media literacy [25]. In the area of content regarding the appearance, this may help one doubt the realism, intention, and ideological purpose behind media images. This can ultimately lessen the internalization of idealized beauty standards and the development of body dissatisfaction [19, 26]. Earlier studies established that media literacy can make a person less vulnerable to social comparison regarding appearance. In addition, media literacy may be associated with lower body dissatisfaction [15, 27]. It may therefore be expected that media literacy can be correlated with body image and also attitudes toward cosmetic surgery. In particular, body image may be one of the key mechanisms through which media literacy works.

Despite the fact that a considerable body of studies has examined the links between social media use, body image, and acceptance of cosmetic surgery, far fewer have investigated media literacy in the same explanatory model. This holds especially for studies that include young women and men in early adulthood [18, 27]. In this sense, the present study aims to analyze the association between media literacy and acceptance of cosmetic surgery among young adults and to test the mediating role of body image in this association.

## Research Hypotheses

H1. Media literacy is significantly associated with body image among young adults.

H2. Media literacy is significantly associated with acceptance of cosmetic surgery among young adults.

H3. Body image is significantly associated with acceptance of cosmetic surgery among young adults.

H4. Body image may play a partial mediating role in the association between media literacy and acceptance of cosmetic surgery.

## Materials and Methods

### Study Design

This study employed a descriptive, cross sectional design. Data were collected online, and the reporting of the study was guided by the STROBE statement for observational research [28].

### Population and Sample

The target population consisted of women and men in Türkiye aged 18–24 years who were literate, had internet access, and agreed to participate voluntarily the final sample comprised 394 young adults who met the inclusion criteria between December 2025 and February 2026.

Participant invitations and the survey link were disseminated through social media platforms, including Facebook, Twitter, and Instagram, as well as messaging applications such as WhatsApp. Data were collected using snowball sampling in an online format. Sample power was calculated using G*Power 3.1.9.7 and was found to be 99% (*d* = 0.44; *α* = 0.05). Inclusion criteria were being between 18 and 24 years of age, being able to read and write Turkish, having internet access, and providing informed consent. Individuals with communication barriers and those unwilling to participate were excluded.

### Data Collection Instruments

Data were collected using the Introductory Information Form, the Body Image Scale, the Acceptance of Cosmetic Surgery Scale, and the Media Literacy Scale.

**Introductory Information Form:** This form included questions on age, gender, educational background, and social media use characteristics.

**Body Image Scale (BIS):** Developed by Saylan and Soyyiğit [29], this five-point Likert-type scale consists of 21 items and four subdimensions: Negative Body Perception, Evaluation Sensitivity, Positive Body Perspective, and Body Change. Items 4, 7, 15, 16, and 17 are reverse-scored. Scores range from 21 to 105, with higher scores indicating a more negative body image. Therefore, higher BIS scores reflect greater body dissatisfaction and more negative body-related perceptions [29]. In the present study, Cronbach’s alpha was 0.91.

**Acceptance of Cosmetic Surgery Scale (ACSS):** Originally developed by Henderson-King and Henderson-King [30] and adapted into Turkish by Karaca et al. [31], this 15-item scale uses a seven-point Likert response format and includes personal, social, and consideration-related dimensions. Higher scores indicate more favorable attitudes toward cosmetic surgery [29, 31]. In this study, Cronbach’s alpha was 0.93.

**Media Literacy Scale (MLS):** Developed by Karaman and Karataş [32], the MLS consists of 17 items and three subdimensions: Being Informed, Ability to Analyze and React, and Ability to Judge and Detect Implicit Messages. Higher scores indicate greater media literacy [32]. In this study, Cronbach’s alpha was 0.96.

### Data Collection Procedure

The data were collected from December 2025 to February 2026. The study was announced through Instagram, Facebook, and Twitter, and participation was voluntary. Study participants were asked to share the link with eligible people in their network.

The survey began with a page that contained details about the research project, its voluntary participation, and confidentiality of the data, along with an electronic informed consent form. Participants were instructed to fill out the questionnaire after giving consent.

Several procedures were used to improve the quality of the data. The survey platform was set up to prevent multiple responses from the same user. The dataset was checked again for the same IP address and very short periods of completion. Responses considered inattentive or invalid were excluded. Moreover, all items on the survey had been set as required, so that incomplete forms could not be submitted.

### Statistical Analysis

The statistical analyses were performed using SPSS 22.0. Descriptive statistics were presented as a number, percentage, mean, and standard deviation. Normality was assessed using the skewness and kurtosis values that fall in the range of ±1.5 [33]. Pearson’s correlation analysis was employed to investigate the associations between MLS, BIS, and ACSS.

In order to assess whether body image mediated the effect of media literacy on acceptance of cosmetic surgery, regression-based mediation testing was performed using PROCESS Macro Model 4 [34]. This model separately tests the effect of the independent variable (X) on the mediator (M) (i.e., path a), the total effect of X on the dependent variable (Y) (i.e., path c), and the effect of X on Y after including M (i.e., path c′). The statistical significance of the indirect effect was examined through bootstrap analysis with 5000 resamples. An indirect effect was considered statistically significant if the 95% confidence interval did not include zero. Statistical significance was set at *p* < .05.

## Results

Of the participants, 76.6% were women, 88.8% were single, 91.4% had university-level education, 76.1% were not employed, and 58.6% reported that their income was lower than their expenditure. With regard to preferred social media content, the most frequently followed category was fashion and beauty (39.2%), followed by news and current affairs (34.4%, *n* = 134), sport and lifestyle (19.3%, *n* = 76), and health and nutrition (6.9%, *n* = 27) (Table 1).

**Table 1:** Distribution of participants according to descriptive characteristics (*n* = 394)

Variables	*n* (%)
*Gender*	
Female	302 (76.6)
Male	92 (23.4)
*Marital status*	
Single	350 (88.8)
Married	44 (11.2)
*Education level*	
High school and below	34 (8.6)
University and above	360 (91.4)
*Employment status*	
Employed	94 (23.9)
Unemployed	300 (76.1)
*Perceived monthly income level*	
Income lower than expenses	231 (58.6)
Income equal to expenses	114 (28.9)
Income higher than expenses	49 (12.4)
*Most frequently followed content type on social media*	
Fashion/beauty	153 (39.2)
News/current affairs	134 (34.4)
Sports/lifestyle	76 (19.3)
Health/nutrition	27 (6.9)
	$$\overline{X} \pm {\mathrm{SD}}$$
Age (years)	22.32±4.47

The mean total score for the Media Literacy Scale was 60.83 ± 15.11, the mean Body Image Scale score was 51.92 ± 14.91, and the mean Acceptance of Cosmetic Surgery Scale score was 50.71 ± 21.40. A weak but statistically significant negative correlation was observed between media literacy and body image (*r* = − .102, *p* < .05). A very weak but significant positive correlation was found between media literacy and acceptance of cosmetic surgery (*r* = .070, *p* < .05). Body image, meanwhile, showed a positive and near-moderate association with acceptance of cosmetic surgery (*r* = .291, *p* < .001) (Table 2).

**Table 2:** Relationships among participants’ total MLS, BIS, and ACSS mean scores (n = 394)

Variables	Mean ± SD	1	2	3
MLS total score (1)	60.83±15.11	–	− .102* .043	.070* .012
BIS total score (2)	51.92±14.91	–	–	.291** .000
ACSS total score (3)	50.71±21.40	–	–	–

*MLS* Media Literacy Scale, *BIS* Body Image Scale, *ACSS* Acceptance of Cosmetic Surgery Scale, $\overline{X}$ Mean, *SD* Standard deviation, *p<0.05, **p<0.01

The mediation analysis demonstrated that media literacy was significantly and negatively associated with body image (*β* = − 0.300, SE = 0.070, *t* = − 4.286, *p* < 0.001). Because higher BIS scores indicate a more negative body image, negative regression coefficients should be interpreted as reflecting lower levels of negative body image perceptions (Table 3).

**Table 3:** Mediating role of body image in the relationship between media literacy and acceptance of cosmetic surgery

Dependent variable	Independent variable	ß	SE	*t*	*p*	95% CI lower	95% CI upper
BIS	MLS (a)	− 0.300	0.070	− 4.286	**0.000**	− 0.437	− 0.163
*R* = 0.32; *R*^2^ = 0.10; *F* = 18.38; *p* = 0.000							
ACSS	MLS (c)	0.280	0.060	4.667	**0.000**	0.162	0.398
*R* = 0.30; *R*^2^ = 0.09; *F* = 16.74; *p* = 0.000							
ACSS	MLS (c′)	0.175	0.070	2.500	**0.013**	0.038	0.312
	BIS (b)	0.350	0.080	4.375	**0.000**	0.193	0.507
*R* = 0.41; *R*^2^ = 0.17; *F* = 22.91; *p* = 0.000							
Total effect	0.280	0.060	4.667	0.000	0.162	0.398	
Direct effect	0.175	0.070	2.500	0.013	0.038	0.312	
Indirect effect	0.105	0.034	–	–	0.038	0.172	

*BIS* Body Image Scale, *MLS* Media Literacy Scale, *ACSS* Acceptance of Cosmetic Surgery Scale, *β* (*B*) standardized regression coefficient, *SE* standard error, *t* *t* value, *p* significance level, *95% CI lower* lower bound of the 95% confidence interval, *95% CI upper* upper bound of the 95% confidence interval, *R* correlation coefficient, *R*^2^ explained variance ratio, *F* overall significance test of the model, path a: Independent variable → mediator effect, path b: Mediator → dependent variable effect, path c: Independent variable → total effect on the dependent variable, path c′: direct effect of the independent variable after inclusion of the mediator in the model

Bold values indicate statistically signifant results (*p* < 0.05)

Media literacy also had a statistically significant total effect on acceptance of cosmetic surgery (*β* = 0.280, SE = 0.060, *t* = 4.667, *p* < 0.001). However, when body image was entered into the model, the direct effect of media literacy on cosmetic surgery acceptance decreased but remained significant (*β* = 0.175, SE = 0.070, *t* = 2.500, *p* = 0.013). This pattern indicates partial mediation.

**Fig. 1:**
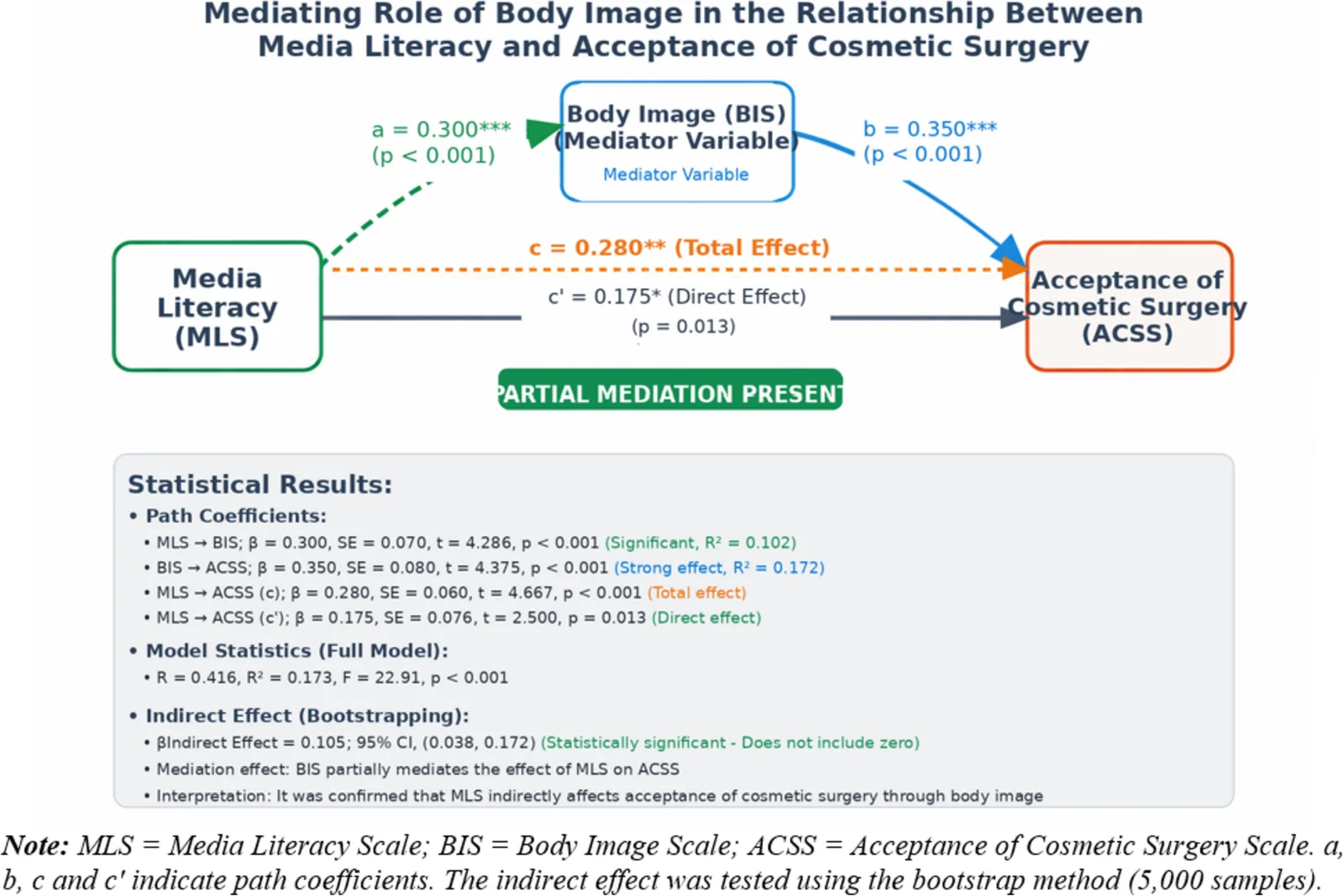
Meditation model illustrating the relationship between media literacy and acceptance of cosmetic surgery through body image

In addition, body image was significantly and positively associated with acceptance of cosmetic surgery (*β* = 0.350, SE = 0.080, *t* = 4.375, *p* < 0.001). Bootstrap analysis showed that the indirect effect was statistically significant, as the 95% confidence interval did not include zero [0.038, 0.172]. Taken together, these findings confirm that body image functions as a significant partial mediator in the relationship between media literacy and acceptance of cosmetic surgery. In other words, media literacy appears to influence cosmetic surgery acceptance both directly and indirectly through body image (Fig. 1).

## Discussion

This research examined the mediating role of body image on the relationship between media literacy and acceptance of cosmetic surgery. The results show that having a higher media literacy correlates with a less negative body image, which was associated with variation in acceptance of cosmetic surgery. The findings suggest that critically engaging with media content may be associated with lower levels of uncritical internalization of digital appearance ideals among young adults.

A slight negative correlation emerges between media literacy and body image, suggesting that having media literacy may allow one to question the belief that media images depict reality, as they may more readily understand that images are manipulated and staged. Such critical engagement may be associated with lower levels of internalization of idealized body standards and appearance comparison, both of which are known to contribute to body dissatisfaction [19, 26, 35, 36]. The relatively small size of the association is not surprising at the same time. Body image is shaped by numerous and interdependent factors, including peer culture, gender norms, familial messages, social conventions, and individual psychological makeup. It is better to think about media literacy as one relevant factor among many, and not as the explanation.

Although several associations reached statistical significance, the observed effect sizes were generally weak. Therefore, these findings should be interpreted with caution in terms of practical significance. Media literacy may represent only one of many factors associated with body image and attitudes toward cosmetic surgery among young adults.

The study found that media literacy and acceptance of cosmetic surgery are closely related, but the association is weak. As it may seem at first glance, this may be counterintuitive, as the general intuition behind media literacy is that it may reduce the persuasive effect of unrealistic beauty standards. Nevertheless, the outcome may indicate a more complicated reality. Young adults who are media literate may also be more involved in digital media environments in general, and therefore more frequently exposed to discussions of cosmetic interventions, aesthetic self-improvement, and appearance-focused trends. So one can develop critical awareness through heightened familiarity. In this way, media literacy may limit naive internalization, but it will not insulate people from the cultural normalization of cosmetic procedures.

The earlier literature is more directly aligned with a more positive association between negative body image and cosmetic surgery acceptance. As people are less satisfied with their body image, they may become more open to surgical alteration to deal with that issue. Selfie activities, editing, filtering, or comparing self-image repeatedly to visually idealized others are likely to enhance dissatisfaction and render aesthetic intervention increasingly thinkable and possibly routine [1, 37]. Evidence also indicates that body dissatisfaction and social appearance anxiety are 2 of the strongest predictors of intent to undergo procedures [38, 39].

The mediation findings suggest that the association between media literacy and attitudes toward cosmetic surgery may be partly explained through body image. Individuals with stronger media literacy skills may be less likely to internalize unrealistic appearance ideals uncritically, which may be associated with a less negative body image and lower acceptance of cosmetic surgery. This is in line with the Tripartite Influence Model, which proposes that sociocultural inputs shape body-related outcomes primarily through internalization and comparison processes. Individuals with stronger media literacy skills may be less likely to absorb unrealistic appearance ideals uncritically; this may be associated with a more stable or less negative body image and lower acceptance of cosmetic surgery. The present findings, therefore, suggest that body image may represent a potentially important associated mechanism between media evaluation skills and aesthetic attitudes.

Taken as a whole, this study contributes to the literature by bringing together media literacy, body image, and cosmetic surgery acceptance within a single analytical model. In particular, the findings regarding the mediating role of body image offer a more refined understanding of how media-related competencies may be associated with aesthetic attitudes. What appears to matter is not only how much media young adults consume, but also how they interpret what they see and how that interpretation feeds into their bodily self-evaluations.

## Conclusion

This study explored the mediating role of body image in the relationship between media literacy and acceptance of cosmetic surgery among young adults. The findings show that higher media literacy is associated with lower levels of negative body image and that body image partially mediates the relationship between media literacy and cosmetic surgery acceptance. Meanwhile, the very weak positive direct association between media literacy and acceptance of cosmetic surgery implies that the relationship is not straightforward and may speak to the complex ways in which awareness, exposure, and sociocultural normalization interact in digital spaces.

From a practical point of view, these findings suggest that interventions that strengthen media literacy may be useful not only to promote critical engagement with media content but also to promote healthier body-related perceptions. Programs aimed at youth may be more effective when they combine media literacy and body image processes rather than treating them as separate components.

## Limitations

The findings of this study must be interpreted while keeping the limitations in mind. The cross sectional design helps to identify relationships, but does not allow causal conclusions. Due to the cross sectional nature of the study, causal inferences cannot be established regarding the relationships among media literacy, body image, and acceptance of cosmetic surgery. Longitudinal or experimental studies could help clarify the causal relationships among media literacy, body image, and acceptance of cosmetic surgery.

In this study, snowball sampling was used to collect data through online self-report questionnaires. As a result, this may have limited the sample’s representativeness and increased the likelihood that sociodemographically similar individuals were overrepresented. Thus, the results should be generalized with care. To begin with, all variables were self-reported. The results may have been impacted by social desirability, subjective interpretation, or response bias.

Ultimately, although the research aimed at young adults with varying sociocultural characteristics, the sample mostly included women and people with a university-level education. It limits the degree to which the results can be generalized to other populations.

The predominance of female and highly educated participants may have limited the representativeness of the sample and reduced the external validity of the findings. In addition, the mediation model was primarily constructed in line with the main aim and hypothesized relationships of the study, focusing specifically on the associations among media literacy, body image, and acceptance of cosmetic surgery. Therefore, potential confounding variables such as gender, social media use characteristics, and socioeconomic status were not controlled for in the mediation analyses. Nevertheless, these variables may have influenced the observed associations.

Future research would be strengthened by a more balanced gender distribution and greater diversity in education and social class. Future studies should also incorporate relevant covariates into mediation models to provide a more comprehensive understanding of the relationships among media literacy, body image, and acceptance of cosmetic surgery.

## Data Availability

The datasets generated and/or analyzed during the current study will be made available by the corresponding author upon request from the editor or reviewers.
